# Description of a new species of *Zeuxevania* Kieffer (Hymenoptera: Evaniidae) from India with a key to species

**DOI:** 10.3897/BDJ.9.e59487

**Published:** 2021-03-10

**Authors:** Sarfrazul Islam Kazmi, Anandhan Rameshkumar

**Affiliations:** 1 Zoological Survey of India, Kolkata, India Zoological Survey of India Kolkata India

**Keywords:** Ensign wasp, *
hubeni
*, Evanioidea, parasitoids, new species, Kerala, *
Zeuxevania
*, India

## Abstract

**Background:**

Taxonomy and diversity of Evaniidae from India have not been studied in depth and hosts of many species are unknown. Out of 31 world genera, only five genera (*Evania* Fab., *Prosevania* Kieffer, *Szepligetella* Bradley, *Vernevania* Huben & Deans and *Zeuxevania* Kieffer) are reported from India. Based on the collection deposited in the Hymenoptera section of Zoological Survey of India (National Zoological Collection), here we are describing a new species of the genus *Zeuxevania*.

**New information:**

A new evaniid species *Zeuxevania hubeni* **sp. nov.** is described, based on a female specimen collected from Kadaludi Bird Sanctuary, Kerala, India. The new species is compared with *Z. curvicarinata* (Cameron), as well as *Z. kasauliensis* (Muzaffer) and a key to Indian species, based on females, is provided. The type specimen is deposited in the National Zoological Collection, Zoological Survey of India, Kolkata, India.

## Introduction

The genus *Zeuxevania* was erected by Kieffer (1902) with *Evania dinarica* Schletterer, 1886 as its type species. Two genera, *Parevania* and *Papatuka* were recently treated as junior synonyms of *Zeuxevania* (Sharanowski et al. 2019). The genus currently consists of 39 world species with four species from India (including three fossils and one species described here) distributed predominantly in the Afrotropical and Oriental region (Deans 2005, Deans et al. 2019, Sharanowski et al. 2019). The species of *Zeuxevania* have been reported as parasitoids of oothecae of Blattellidae (Roth 1985). The present paper deals with the description of a new *Zeuxevania* species from Kerala, India. A key to Indian species of *Zeuxevania*, based on females, is provided.

## Materials and methods

The specimen was collected using a sweep net from Kadaludi Bird Sanctuary, Kerala, India, killed by ethyl acetate and stored in 70% ethyl alcohol. The specimen was later dried and mounted on a rectangular card using water-soluble glue. Photographs were taken with a Nikon DS-Ri2 camera, mounted on a Nikon SMZ25 stereozoom microscope and processed by the NIS-Elements BR Analysis v5.20.00 software. Generic placement was determined using the key provided by Deans and Huben (2003). The examined holotype is deposited in the National Zoological Collection, Zoological Survey of India, Kolkata, India (NZC). The following abbreviations are used in the text: OOL – Minimum distance between the posterior ocellus and eye margin; POL – Minimum distance between the two posterior ocelli; OAL – Minimum distance between the posterior ocellus and anterior ocellus; F1-F11 – Funicular segments 1-11.

## Taxon treatments

### Zeuxevania hubeni

Kazmi and Rameshkumar 2021 sp. n.

D592FE16-DF42-52D3-B1F9-E39517BF79C2

urn:lsid:zoobank.org:act:B0B430AF-BB71-4A9A-B6EC-020DC8A1DB60

#### Materials

- **Type status:** Holotype. **Occurrence:** recordedBy: K. G. Emiliyamma; individualCount: 1; sex: female; lifeStage: adult; **Taxon:** phylum: Arthropoda; class: Insecta; order: Hymenoptera; family: Evaniidae; **Location:** continent: Asia; country: India; countryCode: IN; stateProvince: Kerala; municipality: Kozhikode; locality: Kadaludi Bird Sanctuary; **Identification:** identificationID: Reg. No. 24571/H3; identifiedBy: Kazmi and Rameshkumar; **Event:** eventID: Kadaludi Bird Sanctuary; samplingProtocol: Net Sweep; eventDate: 05 May 2005; habitat: Forest; **Record Level:** institutionID: Zoological Survey of India

#### Description

Female (Fig. 1a, b). Body length 4.2 mm.

*Head*. Black and smooth; ocelli and eyes silvery; genae smooth, lustrous (Fig. 1c, d); mandibles brown with yellow patch, teeth brownish-yellow; antennal radical, scape, pedicel and F1 yellowish; F2 brownish-yellow, remaining antennal segments (F3-F11) dark brown. Head almost quadrate; frontovertex smooth, flat, about half of head width, almost equal to scape length; POL 0.5× OOL, 1.2× OAL; malar space 3.0× POL; maxillary palp 5-segmented; labial palp 3-segmented; mandibles 3-dentate; a pair of keels runs obliquely below the eye to the base of clypeus; antennal sockets arise just above the base of eyes; antenna 13-segmented (Fig. 2a); scape 2.4× longer than broad, subequal to eye length; pedicel 0.5× F1; F1 2.7× as long as broad. Relative measurements (in mm) – head width (height), 0.9 (0.1); frontovertex width, 0.5; POL, 0.1; OAL, 0.08; OOL, 0.19; eye length (width), 0.6 (0.3); malar space length, 0.32; scape length (width), 0.6 (0.25); pedicel length (width), 0.2 (0.12); F1 length (width), 0.4 (0.15).

*Mesosoma*. Orange brown; tegulae testaceous; mid-lobe of mesoscutum dark brown; mesosoma smooth not punctate on dorsal side, foveate on lateral side; mesoscutum 0.7× longer than broad; pronotum with long setae evenly distributed; pronotal neck obscured; dorsal pronotal area concealed medially; notaulus present as continuous furrow, curved towards middle reaching up to posterior margin; parascutal carina present; mesoscutum silvery setae evenly distributed, mid-lobe elevated; parapsidal line present; scutellum 0.5× longer than broad, 0.6× mesoscutum; mesoscutellum smooth with long and stiff setae evenly distributed; transcutal articulation absent; mesepimeral sulcus present; mesopleural pit absent; dorsal propodeal area with long setae (compared with mesoscutum and scutellum) evenly distributed; nucha not elevated.

*Legs* (Fig. 2c): Fore and mid coxa orange brown; fore and mid trochanter whitish-brown; fore and mid femur brown to dark brown; fore tibia and tarsi yellowish-brown; mid-tibia, tarsi brown; hind coxa white except apices brown, not punctate; trochanter white; hind femur dark brown; hind tibia basal 1/3 yellowish-brown, rest of hind legs dark brown; hind tibial spur yellowish; hind tibia as long as hind femur length, 0.6× mesosoma height; external tibial spur 0.75× hind metatarsus; spine present on the dorsal surface of hind tibia. Relative measurements (in mm) – mesosoma height, 1.0; mesoscutum length (width), 0.5 (0.7); scutellum length (width), 0.3 (0.6); hind femur length (width), 1.5 (0.3); hind tibia length (width), 1.5 (0.2); hind metatarsus length, 0.6; tarsal length (1-5), 1.5; interior tibial spur length, 0.25; exterior tibial spur length, 0.45.

*Wings* (Fig. 2b). Hyaline; seven cells; 1RS vein attached with Sc+R at base of stigma; 1M clearly separated from Sc+R; 3M, 3CU present; r-m present as nebulous vein; fore wing 2.5× longer than wide; hind wing 3.0× longer than wide; hamuli consisting of five hooks; M + CU longer than jugal lobe. Relative measurements (in mm) – fore wing length (width), 3.0 (1.2); hind wing length (width), 1.8 (0.6).

*Metasoma* (Fig. 2d). Lustrous brown; ovipositor exserted, 0.2× length of mesosoma; petiole smooth and shiny, basal half dark brown, apical half white, 0.46× metasoma and 3.5× longer than broad; ovipositor exserted, 0.5× petiole length. Relative measurements (in mm) – petiole length (width), 0.7 (0.2); metasoma length (width), 1.5 (1.2); ovipositor (exserted part) length, 0.35.

#### Etymology

The species is named after Mr. M. Huben, Museum of Comparative Zoology, Harvard University, Cambridge, MA, USA in recognition of his contribution to the taxonomy of Evanioidea.

#### Distribution

India: Kerala.

#### Taxon discussion

*Zeuxevania hubeni* sp. nov. is very close to *Z. curvicarinata* (Cameron) (Cameron 1899), but differs in having brown mandibles with yellow patch; mesosoma orange brown, hind coxa white, except brown apices without punctures; hind tibia basal 1/3 yellowish-brown; metatarsus dark brown; petiole smooth and shiny, longer spur of hind tibia 0.75× metatarsus length (in *Z. curvicarinata*, mandibles reddish; mesosoma red; hind coxa black, transversely punctuate, more strongly at the base than apex; hind tibia and metatarsus white; petiole with few large punctures; longer spur of hind tibia nearly equal to the length of metatarsus). In comparison with *Z. kasauliensis* (Mani and Muzaffer 1943), it differs in the following features: mandibles brown with yellow patch; the base of antennae yellowish; eye length and width ratio 2:1; hind coxa not punctate; basal 1/3 hind tibia yellowish-brown; hamuli consisting of five hooks; petiole basal half dark brown, apical half white (in *Z. kasauliensis*, mandibles reddish-brown; antennae dark reddish-brown; eye length and width ratio 10:7; hind coxa punctuate; hind tibia darker; hamuli consisting of nine hooks; petiole dark brown).

## Identification Keys

### Key to Indian species of Zeuxevania Kieffer (Based on females)

**Table:** 

1	Face without keels (malar sulcus) [petiole distally white; thorax and base of flagellum black]	*albitarsis* (Cameron)
–	Face with an oblique keel (the malar sulcus) running from below each eye to the base of clypeus	2
2	Petiole black; antenna reddish-brown [hind tibia and tarsus spinose]	*kasauliensis* (Muzaffer)
–	Petiole with basal half dark brown, apical half white; basal segments of antenna yellowish or white	3
3	Hind coxa white without punctures; mesosoma orange-brown; the base of flagellum yellowish; petiole without punctures	*hubeni* Kazmi & Rameshkumar sp. nov.
–	Hind coxa black with transverse punctures; mesosoma red; the base of flagellum white; petiole with few large punctures	*curvicarinata* (Cameron)

## Supplementary Material

XML Treatment for Zeuxevania hubeni

## Figures and Tables

**Figure 1a.**
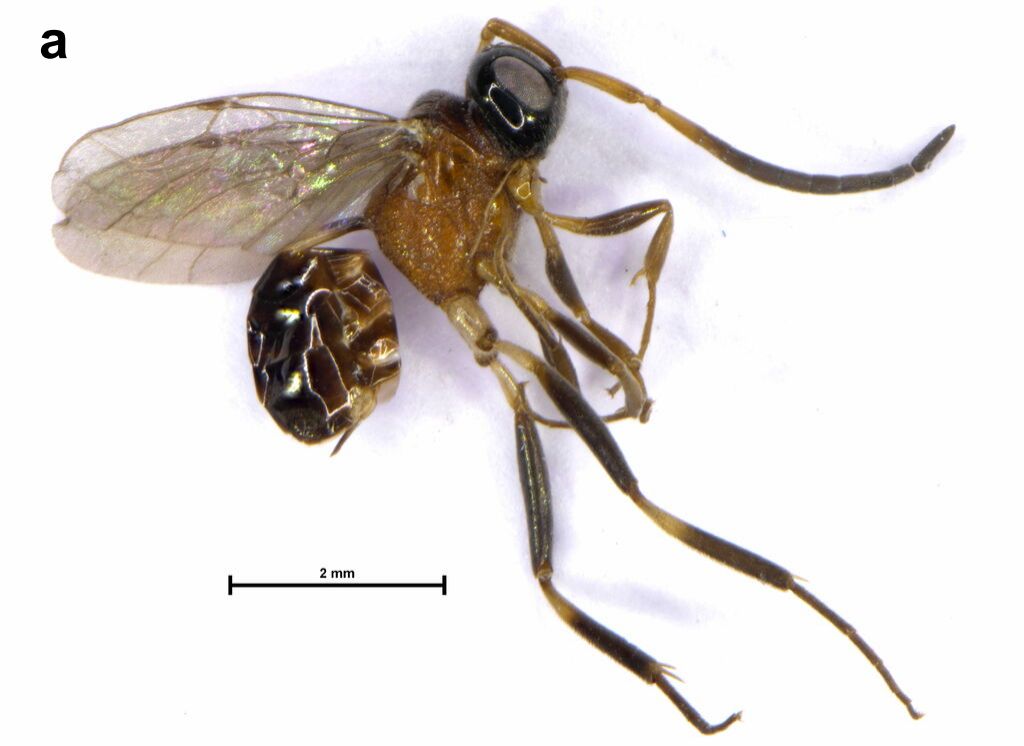
Habitus

**Figure 1b.**
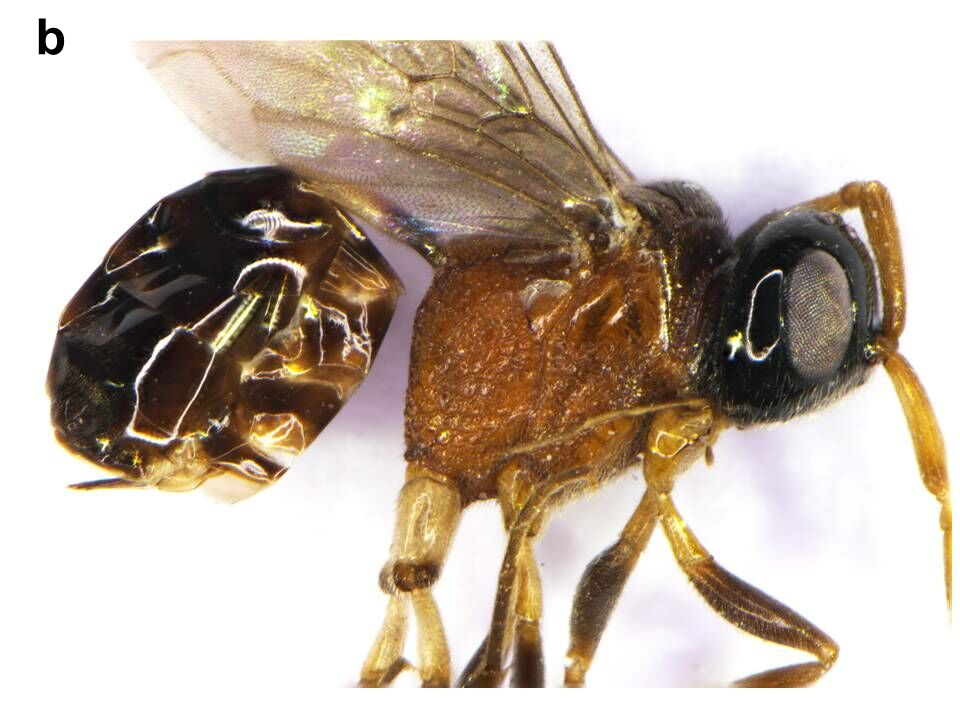
Head, meso and metasoma, lateral view

**Figure 1c.**
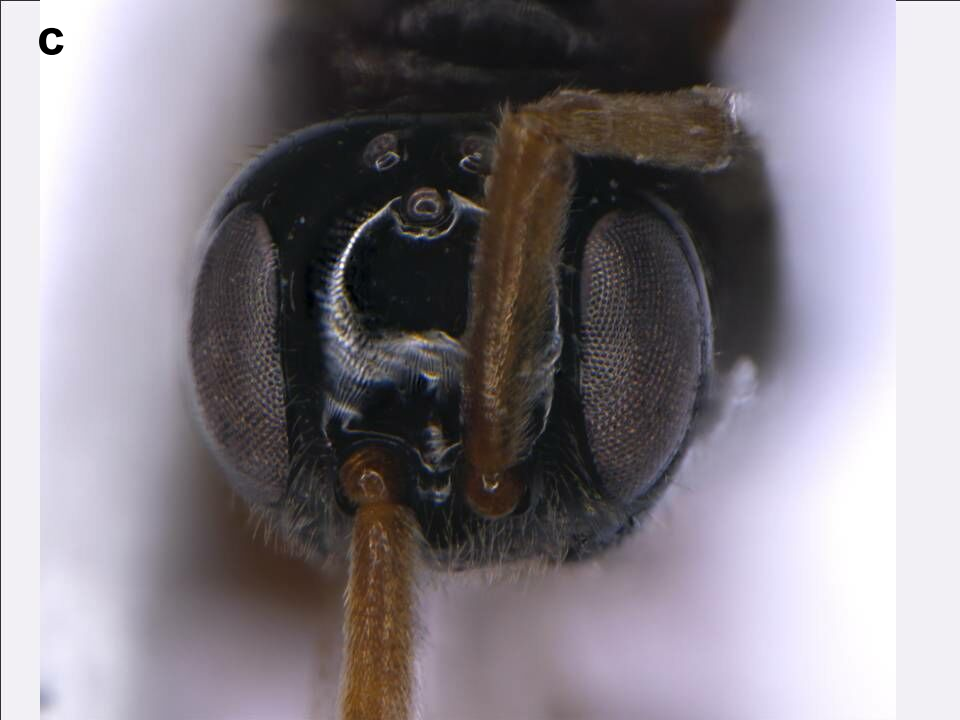
Head frontal upper view

**Figure 1d.**
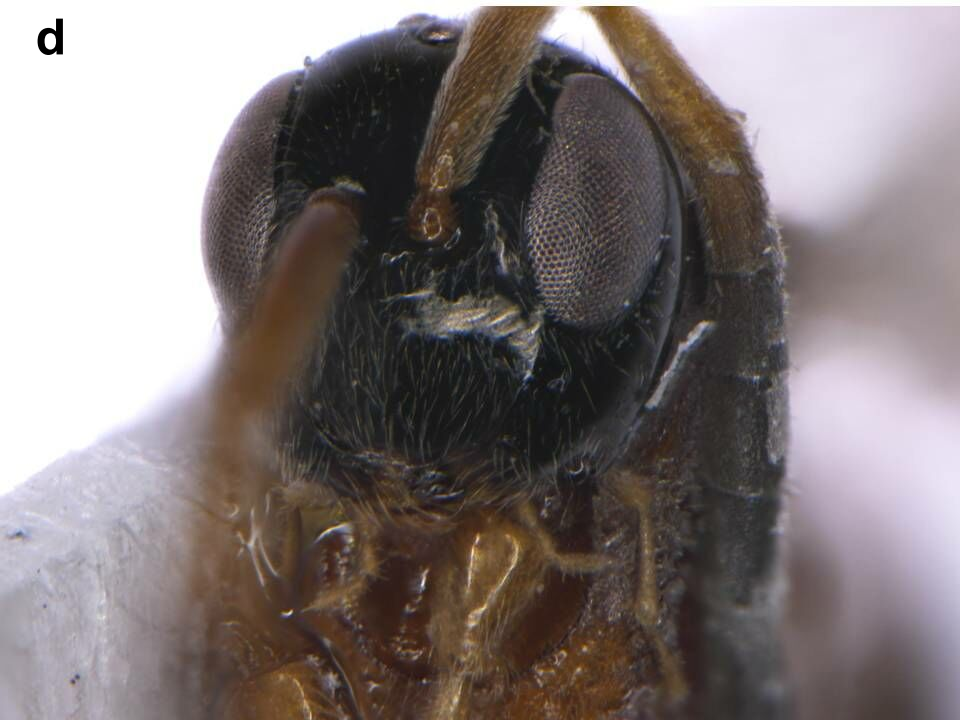
Head frontal lower view

**Figure 2a.**
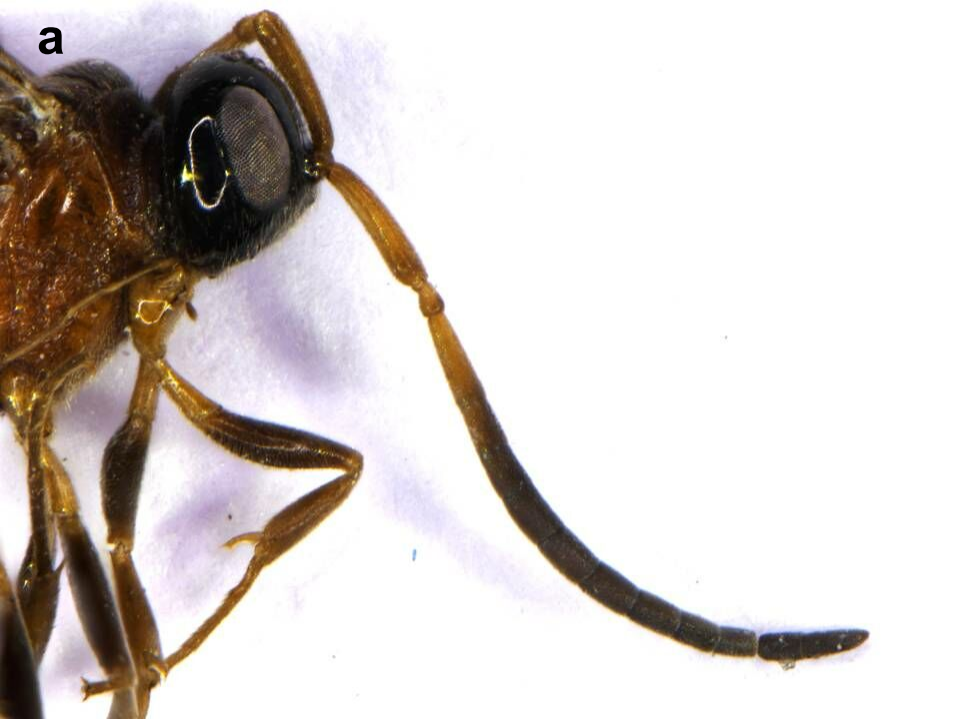
Antenna

**Figure 2b.**
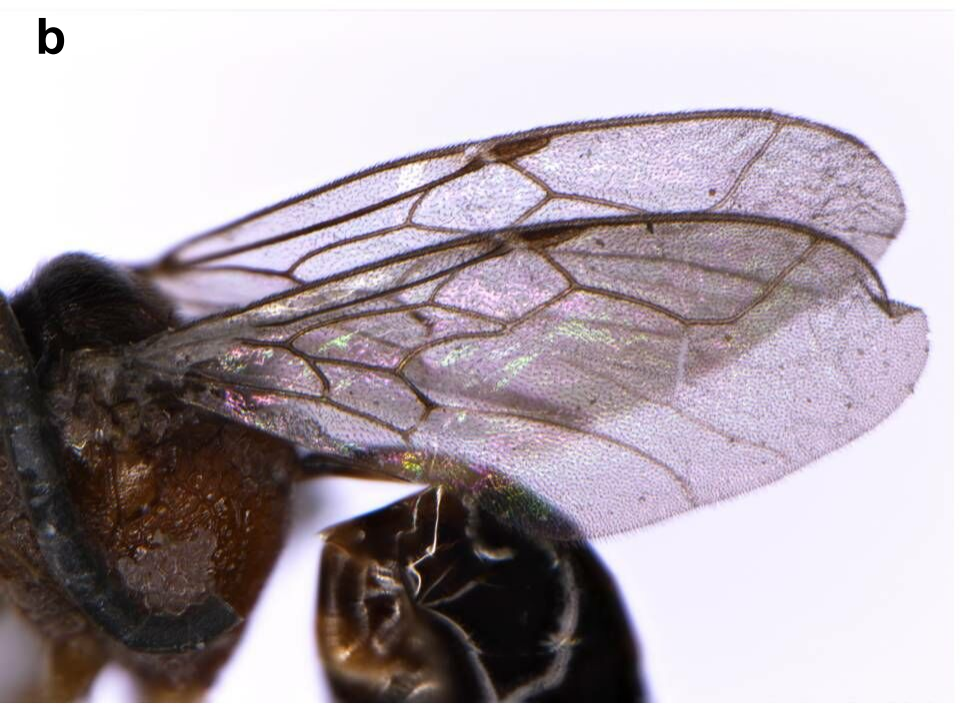
Fore wing

**Figure 2c.**
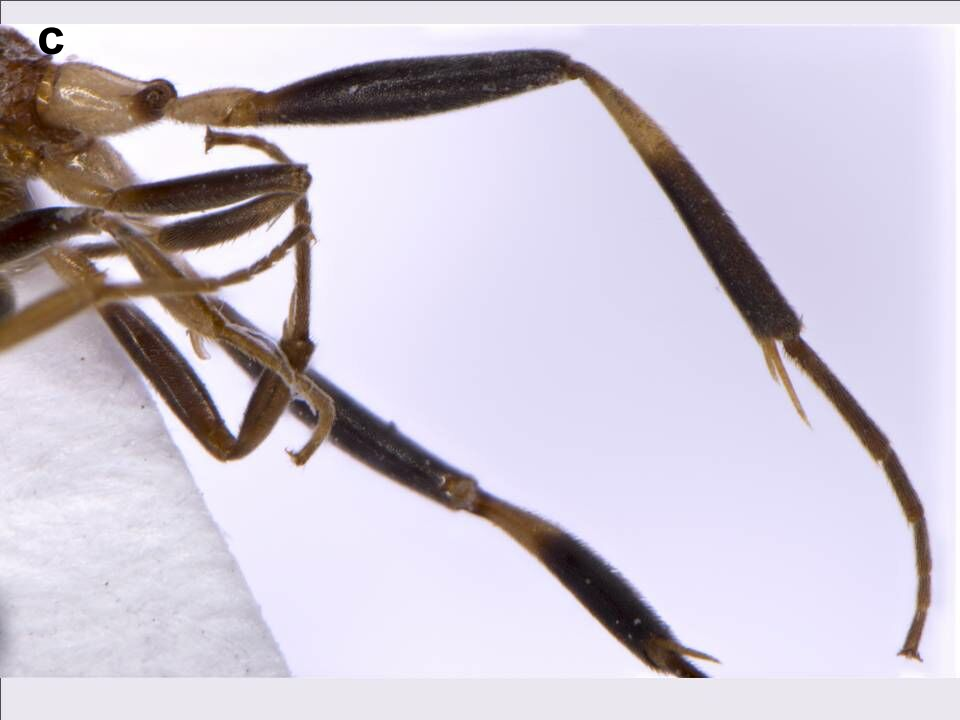
Hind leg

**Figure 2d.**
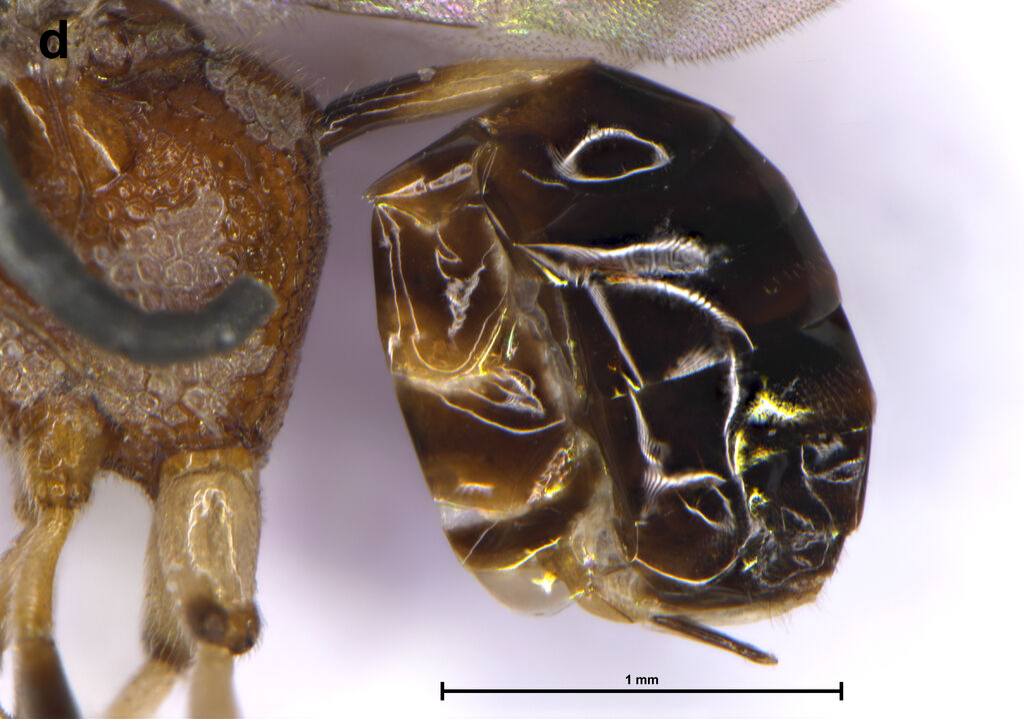
Metasoma with petiole
